# Percolated Sulfide in Salt‐Concentrated Polymer Matrices Extricating High‐Voltage All‐Solid‐State Lithium‐metal Batteries

**DOI:** 10.1002/advs.202202474

**Published:** 2022-06-24

**Authors:** Feng Jiang, Yantao Wang, Jiangwei Ju, Qian Zhou, Longfei Cui, Jinzhi Wang, Guoxi Zhu, Huancheng Miao, Xinhong Zhou, Guanglei Cui

**Affiliations:** ^1^ College of Chemistry and Molecular Engineering Qingdao University of Science and Technology Qingdao 266042 P. R. China; ^2^ Qingdao Industrial Energy Storage Research Institute Qingdao Institute of Bioenergy and Bioprocess Technology Chinese Academy of Sciences Qingdao 266101 P. R. China; ^3^ School of Future Technology University of Chinese Academy of Sciences Beijing 100049 P. R. China

**Keywords:** all‐solid‐state batteries, high ionic conductivity, high voltage, interfacial compatibility, solid electrolyte

## Abstract

All‐solid‐state lithium‐metal batteries (ASLMBs) are considered to be remarkably promising energy storage devices owing to their high safety and energy density. However, the limitations of current solid electrolytes in oxidation stability and ion transport properties have emerged as fundamental barriers in practical applications. Herein, a novel solid electrolyte is presented by in situ polymerization of salt‐concentrated poly(ethylene glycol) diglycidyl ether (PEGDE) implanted with a three‐dimensional porous L_10_GeP_2_S_12_ skeleton to mitigate these issues. The poly(PEGDE) endows more oxygen atoms to coordinate with Li^+^, significantly lowering its highest occupied molecular orbital level. As a consequence, the electro‐oxidation resistance of poly(PEGDE) exceeds 4.7 V versus Li^+^/Li. Simultaneously, the three‐dimensonal porous L_10_GeP_2_S_12_ skeleton provides a percolated pathway for rapid Li^+^ migration, ensuring a sufficient ionic conductivity of 7.7 × 10^−4^ S cm^−1^ at room temperature. As the bottlenecks are well solved, 4.5 V LiNi_0.8_Mn_0.1_Co_0.1_O_2_‐based ASLMBs present fantastic cycle performance over 200 cycles with an average Coulombic efficiency exceeding 99.6% at room temperature.

## Graphical abstract

A three‐dimensonal composite electrolyte is presented by in situ polymerizing salt‐concentrated polymer implanted with a porous sulfide skeleton. The high‐salt concentration allows more oxygen atoms to coordinate with ions thus significantly enhancing the oxidative stability of the polymer, while the sulfide skeleton provides continuous pathways for fast Li+ transportation. This synergy enables stable cycling of 4.5 V all‐solid‐state lithium‐metal batteries at room temperature.

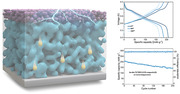

## 1 Introduction

The continuous development of electronic devices puts forward higher demands on the energy density of the battery, which is closely related to the operating voltage.^[^ ^1^, ^2^ ^]^ However, more energy storage in the limited space of lithium batteries always brings more and more safety hazards.^[^ ^3^ ^]^ Unfortunately, the traditional liquid carbonate electrolytes, which are flammable and volatile, trigger severe potential safety concerns for lithium batteries. When liquid electrolytes are substituted by solid electrolytes, whether polymers or inorganics, the safety risks can significantly be lowered.^[^ ^4^, ^5^ ^]^ Therefore, the development of all‐solid‐state lithium‐metal batteries (ASLMBs) is one of the ultimate solutions to address the safety issue as well as the energy density of the battery.

A highly conductive solid electrolyte with good oxidation stability is a key component to realize the promise of ASLMBs.^[^ ^6^, ^7^ ^]^ To ensure satisfactory electrochemical performance, the Li^+^ conductivity ($\sigma_{{\mathrm{Li}}^{+}}$) of the electrolyte must be at least 10^−4^ S cm^−1^.^[^ ^8^ ^]^ In this regard, inorganic electrolytes have more significant advantages than polymeric electrolytes, and some sulfides exhibit comparable or even higher $\sigma_{{\mathrm{Li}}^{+}}$ than liquid electrolytes, such as Li_6_PS_5_Cl (2.04 × 10^−3^ S cm^−1^) and Li_10_GeP_2_S_12_ (1.2 × 10^−2^ S cm^−1^).^[^ ^9^, ^10^ ^]^ However, the inferior solid‐solid contact with electrodes originating from high rigidity pronouncedly increases the interfacial resistance, causing poor battery performance.^[^ ^11^ ^]^ In contrast, the characteristics of flexible geometry and easy processability make polymer electrolytes suitable for battery fabrication and large‐scale production.^[^ ^12^, ^13^ ^]^ Furthermore, relying on the advantages of relatively soft nature and superior adhesion, the polymer electrolytes have decent interfacial compatibility with electrodes.^[^ ^14^ ^]^ Inspired by this, an in situ polymerization strategy is reported to allow the polymer to preserve the compact interfacial properties created by the penetration of liquid monomer precursors, further integrating multiple solid–solid interface at essentially all length scales.^[^ ^15^, ^16^, ^17^, ^18^ ^]^

Nevertheless, the bottlenecks of polymer electrolytes, including insufficient $\sigma_{{\mathrm{Li}}^{+}}$ and limited oxidation stability, hindering their further commercialization in future lithium batteries applications.^[^ ^19^, ^20^, ^21^, ^22^ ^]^ This issue is even more pronounced in the pursuit of high energy density systems while employing high voltage cathodes.^[^ ^19^ ^]^ To resolve the problem of low $\sigma_{{\mathrm{Li}}^{+}}$ owing to the comparatively slow motion of polymer side chains,^[^ ^23^ ^]^ researchers embed inorganic fillers into the polymer matrix to adjust the ionic transportability.^[^ ^24^, ^25^, ^26^, ^27^ ^]^ These filler materials both reduce the crystallinity of the polymer matrix and favor additional ion diffusion paths, which have the possibility to increase the overall $\sigma_{{\mathrm{Li}}^{+}}$ by at least one order of magnitude. On the other hand, (poly(ethylene oxide), PEO)‐based solid electrolytes, which are commercially available polymer electrolytes, can only operate with the cutoff voltage of less than 4 V due to structural limitations.^[^ ^28^ ^]^ Therefore, broadening the electrochemical window of PEO‐based polymer is of great significance for the realization of high‐energy‐density ASLMBs. Researchers have demonstrated that the ether oxygen (EO) segments in glyme donate lone pairs of electrons to the Li^+^ cation, thus favoring to enhance the oxidation stability of the polymer.^[^ ^29^ ^]^ Following that, it is reported that Wu et al. successfully extended the electrochemical window of the PEO electrolyte system to 4.5 V by adopting the polymer‐in‐salt electrolyte strategy.^[^ ^30^ ^]^

**Figure 1:**
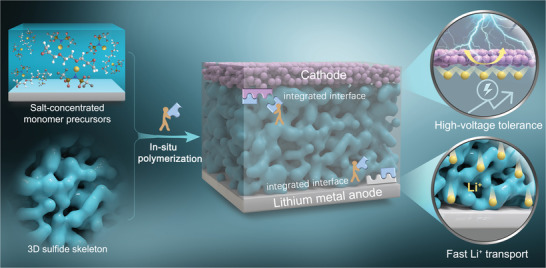
Schematic illustration of the ASLMB with 3D composite electrolyte.

In this work, poly(ethylene glycol) diglycidyl ether (PEGDE), as the as‐designed monomers for in situ polymerization, exhibits excellent solubility for lithium bis(trifluoromethanesulfonyl)imide (LiTFSI). Moreover, the antioxidant capacity of polymers (poly(PEGDE), P(PEGDE)) formed from monomers increases with the concentration of LiTFSI. The electrochemical window is broadened to 4.7 V when the LiTFSI concentration reaches 60 wt %. As verified by classical molecular dynamics simulations, more EO segments coordinate with lithium ions, thus drastically lowering the highest occupied molecular orbital (HOMO) energy level of the polymer. Whereas, experimental data suggest that the higher oxidation potential comes at the expense of a decrease in $\sigma_{{\mathrm{Li}}^{+}}$. In order to simultaneously meet the demands of high‐energy‐density ASLMBs for wide electrochemical window and high $\sigma_{{\mathrm{Li}}^{+}}$, we propose a synergistic strategy, that is, to implant porous Li_10_GeP_2_S_12_ skeleton (p‐LGPS) prepared by sublimation of selenium disulfide at high temperature into the salt‐concentrated P(PEGDE). As illustrated in **Figure** **1**, salt‐concentrated polymer matrix not only achieves high‐voltage tolerance, but also further integrates the electrolyte/electrode interface via in situ polymerization. Meanwhile, the three‐dimensonal (3D) porous skeleton builds a continuous fast channel for the transport of lithium ions, thus greatly improving the $\sigma_{{\mathrm{Li}}^{+}}$ of the electrolyte.^[^ ^31^, ^32^, ^33^ ^]^

Based on the optimal design intentions, an innovative composite electrolyte with a wide electrochemically stable voltage window (5 V) and superior $\sigma_{{\mathrm{Li}}^{+}}$ (7.7 × 10^−4^ S cm^−1^), as well as compact interfacial contact, is successfully prepared. At last but not least, utilizing the protection of the polymer to the sulfide phase, the P(PEGDE)/p‐LGPS composite (3D composite) is perfectly compatible with lithium metal. As a result, the room temperature performance of ASLMBs is completely activated with the help of the synergy, where the as‐designed LiNi_0.8_Mn_0.1_Co_0.1_O_2_ (NCM811)|Li battery based on the electrolyte delivers an outstanding discharge capacity, eminent rate performance and a cycle life of up to 200 cycles at a cut‐off voltage of 4.5 V. The present study opens up a new avenue in boosting the development of all‐solid‐state batteries which also takes a crucial step toward the rational utilization of high‐energy‐density lithium metal batteries.

## 2 Results and Discussion

### 2.1 Effectiveness of Salt‐Concentrated P(PGEDE)

**Figure 2:**
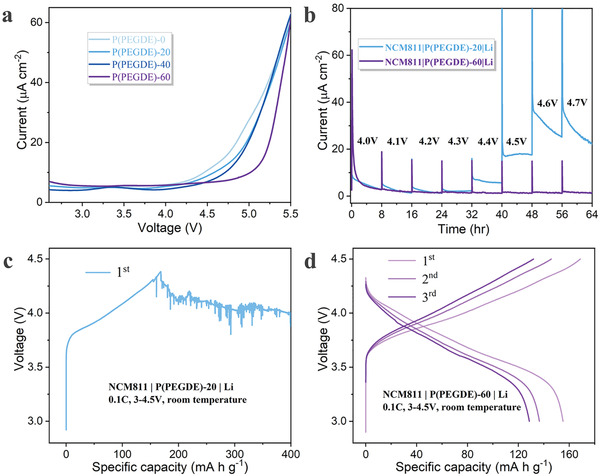
Oxidation behavior of P(PEGDE). a) LSV profiles of P(PEGDE) with varied LiTFSI concentrations at a scan rate of 0.1 mV s^−1^. b) Electrochemical floating analysis of P(PEGDE)‐20 and P(PEGDE)‐60 in the as‐assembled NCM811|Li batteries. Typical charge‐discharge curves of NCM811|Li batteries based on c) P(PEGDE)‐20 and d) P(PEGDE)‐60.

Liquid PEGDE can be converted into solid‐state polymer electrolyte via in situ polymerization triggered by lithium difluoro(oxalate)borate. The oxidation stability of P(PEGDE) with different LiTFSI contents (20 wt %, 40 wt %, 60 wt %) is firstly analyzed by linear scan voltammetry (LSV). For convenience, P(PEGDE) with 20 wt %, 40 wt %, 60 wt % LiTFSI is referred to as P(PEGDE)‐20, P(PEGDE)‐40, and P(PEGDE)‐60 respectively, and additional information can be found in Table S1, Supporting Information. As shown in **Figure** **2**a, the response current of P(PEGDE)‐20 increases gradually at 4.4 V versus Li^+^/Li, indicating oxidation reaction starts and the polymer is decomposed at this voltage. For P(PEGDE)‐40, a significant increase in current is observed from 4.5 V onwards. As the LiTFSI concentration increases to 60 wt %, the decomposition voltage of P(PEGDE) exceeds 4.7 V. It can be tentatively determined that the increase of LiTFSI content gradually enhances the antioxidant properties of P(PEGDE).

Then, P(PEGDE) with various LiTFSI concentrations is evaluated practically in high voltage lithium metal batteries with NCM811 cathode. The electrochemical floating analysis (Figure 2b) provides a more stringent test of the oxidative stability. Monitoring the leakage current obtained at each voltage allows a direct assessment of the importance of electrochemical degradation in a fully charged state.^[^ ^34^ ^]^ In this experiment, NCM811|Li batteries are charged at the voltage range of 4.0–4.7 V in a stepwise ramp and the voltage is maintained at a targeted value for a period of 8 h. The results show that the P(PEGDE)‐60‐based high‐voltage battery is maintained stably above 4.7 V compared to the P(PEGDE)‐20‐based one, which shows a large current leakage at 4.4 V. It is indicated that P(PEGDE)‐60 possesses superior high‐voltage tolerance over P(PEGDE)‐20 at potentials above 4.7 V, which is in full accordance with the LSV results. After the test, both cells are disassembled and Fourier transform infrared spectroscopy (FTIR) is conducted to analyze the molecular structure of P(PEGDE) near the cathode side. For P(PEGDE)‐20, a new characteristic peak belonging to the ester species emerges at 1725 cm^−1^ (Figure S1a, Supporting Information), which can be attributed to the oxidation products of the polymer at high voltage.^[^ ^35^ ^]^ By contrast, for P(PEGDE)‐60, no change in the FTIR spectra is observed (Figure S1b, Supporting Information), suggesting high concentration keeps P(PEGDE) from decomposing even at 4.7 V.

The above two batteries are tested for charging and discharging at a cutoff voltage of 4.5 V. As shown in Figure 2c, when charging, the P(PEGDE)‐20 based NCM811|Li battery suffers from severe voltage fluctuations and cannot reach the cutoff voltage of 4.5 V due to the continuous decomposition. By contrast, the P(PEGDE)‐60 based battery conducts typical charge and discharge behavior (Figure 2d). It is noteworthy that even at a low current density of 0.1 C, the P(PEGDE)‐60 based battery fails in discharge capacity and cycling performance, which is caused by the sluggish ion transport. $\sigma_{{\mathrm{Li}}^{+}}$ of P(PEGDE) at different LTFSI concentrations is calculated by electrochemical impedance spectroscopy (EIS) (Figure S2, Supporting Information). The results show that $\sigma_{{\mathrm{Li}}^{+}}$ of P(PEGDE)‐60 is estimated to be 4.03 × 10^−6^ S cm^−1^ at room temperature, only one‐sixth of that of P(PEGDE)‐20, which cannot meet the working requirements of high energy density batteries.

### 2.2 Mechanism of the Oxidative‐Stability Enhancement

**Figure 3:**
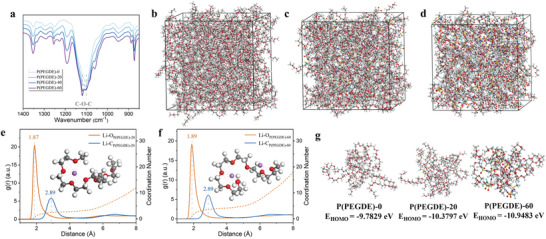
a) FTIR spectra of P(PEGDE) at different LiTFSI concentrations. Snapshots of the final configurations of b) P(PEGDE)‐0, c) P(PEGDE)‐20, and d) P(PEGDE)‐60. The RDF (solid lines) and CN plots (broken lines) of e) P(PEGDE)‐20 and f) P(PEGDE)‐60 from MD simulation trajectories. g) Typical configurations and HOMO energy levels of P(PEGDE) at different LiTFSI concentrations. Color code: C, silver; O, red; H, white; Li, purple; N, blue; S, yellow; F, light blue.

To illustrate the enhanced oxidation stability of P(PEGDE), FTIR is also conducted to analyze the functional group structure evolution with different LiTFSI concentrations. As shown in **Figure** **3**a, the characteristic peak belonging to the EO segments in P(PEGDE) gradually shifts from 1103 to 1120 cm^−1^ with increasing LiTFSI concentration, which can be attributed to the chemical environment change of the C—O—C group. The enhancement of other peaks is caused by the increase of LiTFSI. To further elucidate the specific mechanism of the interaction between LiTFSI and P(PEGDE), molecular dynamics (MD) simulations are performed. Figure 3b,c,d show snapshots of the final configurations obtained from the MD simulations of P(PEGDE), P(PEGDE)‐20, and P(PEGDE)‐60, respectively. According to the MD simulations, the radial distribution functions (RDF) and coordination numbers (CN) between Li ions and EO segments in P(PEGDE) are calculated (Figure 3e,f). For RDF in P(PEGDE)‐20, two sharp peaks corresponding to the Li‐O_P(PEGDE)‐20_ and Li‐C_P(PEGDE)‐20_ are identified at 1.87 and 2.89 Å, which implies complexation occurs between Li ions and EO segments in P(PEGDE). A similar distribution is observed in P(PEGDE)‐60, with strong peaks belonging to Li‐O_P(PEGDE)‐60_ and Li‐C_P(PEGDE)‐60_ at 1.89 and 2.89 Å, respectively. The agreement on the peak positions indicates that the coordination of lithium ions with EO segments is unaffected by increased LiTFSI concentration. In other words, additional lithium ions coordinate with the EO segment in the same way. Furthermore, the CN of Li with O increases from 2.8 to 4.2 with the increase of LiTFSI concentration from 20 to 60 wt %, agreeing well with the conclusion conveyed by the radial distribution function. It is determined that these EO segments coordinate with all lithium ions after accommodating more lithium salts, forming a more plentiful coordination environment.^[^ ^36^ ^]^ Meanwhile, RDF of EO segments with the other ions in LiTFSI (Figure S3, Supporting Information) reveal no closer distribution. The scattered distribution as shown illustrates TFSI^−^ neither correlates with the P(PEGDE) nor affects the chemical environment of P(PEGDE). Then select the typical structure of each solution from the stable MD simulation trajectory to optimize all the structures and calculate the orbital energy gap. Figure 3g shows the HOMO energy level corresponding to different structures according to the calculation. The HOMO energy level of P(PEGDE) without the participation of other substances is −9.7829 eV, drops to −10.3797 and −10.9438 eV after 20 and 60 wt % LiTFSI addition respectively. In light of the foregoing evidence, it is conceivable that the EO segments provide lone pair electrons to the lithium ions, forming a tight coordination relationship. The continuous addition of LiTFSI riches the coordination environment of P(PEGDE), resulting in a lower HOMO energy level. Since electrons are extracted from the HOMO when electrochemical oxidation occurs, a lower HOMO level implies stronger oxidation resistance.^[^ ^37^ ^]^

### 2.3 Implantation of the p‐LGPS Skeleton

**Figure 4:**
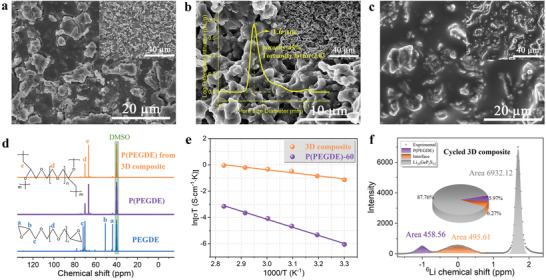
Characterization of p‐LGPS and the 3D composite. a) typical SEM view of pristine SeS_2_+LGPS pellet. b) typical SEM cross‐sectional view of p‐LGPS and pore size distribution of p‐LGPS. c) typical SEM cross‐sectional view of the 3D composite. Inset shows the cross‐sectional view at low magnification. d) ^13^C NMR spectra of PEGDE, pure P(PEGDE), and P(PEGDE) from the 3D composite using deuterated dimethyl sulfoxide as solvent. Their corresponding structural formulas of PEGDE and P(PEGDE) are shown. e) ionic conductivity dependence on temperature of the 3D composite and P(PEGDE)‐60. f) Quantitative fitting of the 3D composite ^6^Li SSNMR spectra after polarization.

The method of 3D p‐LGPS skeleton implantation is adopted to cope with the low $\sigma_{{\mathrm{Li}}^{+}}$ of the polymer caused by the excessive addition of LiTFSI. To prepare the p‐LGPS skeleton, the LGPS is thoroughly mixed with the porogenic agent, namely SeS_2_, and pressed into a pellet. As shown in **Figure** **4**a, scanning electron microscope (SEM) images of the cold‐pressed mixture pellet demonstrate a dense microstructure with SeS_2_ uniform distribution. After thermal treatment at 500 °C for 5 h, SeS_2_ is volatilized to leave a self‐supported porous skeleton with average pore diameter of 1.6 µm, porosity of 45%, and pore tortuosity of 2.03 determined by a mercury intrusion method (Figure 4b). From the energy dispersive spectroscopy (EDS) mapping images (Figure S4, Supporting Information) of the p‐LGPS, it can be clearly observed that all the SeS_2_ has been completely removed during high‐temperature sintering. The resultant porous structure is further revealed via 3D reconstructed images using X‐ray computed tomography technology (Figure S5, Supporting Information). Moreover, the X‐ray diffraction (XRD, Figure S6, Supporting Information) results suggest the XRD pattern of p‐LGPS is consistent well with that of the as‐prepared LGPS powders, indicating no reaction happens between LGPS and SeS_2_. According to the EIS, the $\sigma_{{\mathrm{Li}}^{+}}$ of p‐LGPS at room temperature is estimated to be 4.8 × 10^−4^ S cm^−1^ (Figure S7, Supporting Information). Though this value is 19 times smaller than that of the dense LGPS (9.32 mS cm^−1^) due to the large porosity of p‐LGPS, it is much higher than those of the vogue oxide electrolyte, for example, Li_7_La_3_Zr_2_O_12_ of 10^−4^ S cm^−1^, and polymer electrolyte, for example, PEO of 10^−6^ S cm^−1^.^[^ ^38^, ^39^ ^]^ The adequate porosity, large pore size, and high $\sigma_{{\mathrm{Li}}^{+}}$ suggests p‐LGPS particularly promising porous skeleton for liquid monomers infusion and in situ polymerization.

The PEGDE monomers implanted with p‐LGPS are heat‐treated at 80 °C for 24 h, and the cross‐sectional SEM images vividly show the specific inner structures in the 3D composite. In Figure 4c, the influx and in situ polymerization of monomers resulted in a tight encapsulation of p‐LGPS by the polymer, ensuring intimate contact between the two phases. Besides, the EDS mapping (Figure S8, Supporting Information) confirms the homogeneous distribution of both organic and inorganic components in the 3D composite. Then, the characterization of the molecular structure using nuclear magnetic resonance (NMR) and FTIR further investigated the polymerization state of the monomers in the composite. The ^13^C NMR spectra (Figure 4d) demonstrate that, after thermal treatment at 80 °C for 24 h, the peaks at 43.8 and 50.7 ppm associated with carbon atoms in the epoxy ethyl group marked by a and b vanish entirely as does the peak of the carbon atom near the group marked by c. Instead, a new signal appears at 66.8 ppm, representing the product formed by the polymerization of epoxy groups. Similarly, there is evidence of the successful polymerization of PEGDE in the ^1^H NMR spectra (Figure S9a, Supporting Information), which indicates that the p‐LGPS does not hinder the polymerization of the monomers. Moreover, the FTIR spectra reflected in Figure S9b, Supporting Information, also provide valuable information regarding this in situ polymerization. Concretely, the epoxy‐functional group at 912 cm^−1^ in PEGDE disappears completely after thermal treatment, while the C—O—C at 1100 cm^−1^ remains, proving the transformation from monomers to P(PEGDE). Furthermore, the identical consistency of spectra from 3D composite and pure P(PEGDE) validate that p‐LGPS does not affect the polymerization of PGEDE and no side reactions occur either with the monomers or with the polymer.

To determine the $\sigma_{{\mathrm{Li}}^{+}}$ of the 3D composite, the ohmic resistance (*R*_ohm_) evolution of a steel|steel symmetric cell changing with time at 80 °C is supervised. As shown in Figure S10, Supporting Information, the *R*_ohm_ of the battery grows significantly from 207 to 1208 Ω cm in the first 24 h due to the continuous polymerization of PEGDE to increase the energy barrier for Li^+^ migration. Thereafter, *R*_ohm_ maintains in 1300 Ω cm, reflecting the complete accomplishment of the polymerization. Then, the heat‐treated symmetric cells are continued to be tested for electrochemical impedance at various temperatures. Based on the *R*_ohm_, the $\sigma_{{\mathrm{Li}}^{+}}$ of 3D composite is 7.7 × 10^−4^ S cm^−1^ at room temperature (Figure 4e), which is 191 times higher than that of P(PEGDE)‐60, 4.03 × 10^−6^ S cm^−1^. Additionally, cyclic voltammetry is used to investigate the decomposition voltage of the composite electrolyte. When the voltage is swept to 5 V, as indicated in Figure S11, Supporting Information, there is still no increase in response current, which proves the oxidation potential of the composite electrolyte reaches more than 5 V versus Li^+^/Li. To sum up, the extremely high $\sigma_{{\mathrm{Li}}^{+}}$ and wide electrochemical window indicates that the 3D composite electrolyte is completely suitable for high‐voltage cathodes.

The Li^+^ migration behavior in 3D composite electrolyte is elucidated by solid‐state NMR (SSNMR) techniques using an isotope‐replacement method with ^6^Li as the lithium source.^[^ ^40^, ^41^, ^42^ ^]^ SSNMR is firstly performed on pristine P(PEGDE)‐60 and LGPS to provide a reference for the characteristic peaks of the ^6^Li signal in different local environments. The results (Figure S12, Supporting Information) show the ^6^Li resonance signal of P(PEGDE) is at −1.03ppm, while that of LGPS is at 1.68 ppm. For the pristine 3D composite electrolyte (Figure S13, Supporting Information), besides the above two peaks, a new resonance peak appears at 0.15 ppm, which is the result of the resonance of ^6^Li in the interface phase between LGPS and P(PEGDE).^[^ ^32^ ^]^ Further quantitative analysis based on the spectral fitting shows that the ^6^Li peak areas of the pristine composite in the P(PEGDE) phase, interface phase, and LGPS phase are 123.2, 30.16, and 832.46, respectively. After polarization in ^6^Li|^6^Li symmetric cell, as shown in Figure 4f, the corresponding ^6^Li peak areas are 458.56, 495.61, and 6932.12, in that order. Obviously, the high increase of ^6^Li content in the LGPS phase compared to the polymer phase and the interfacial phase indicates the p‐LGPS with $\sigma_{{\mathrm{Li}}^{+}}$ provides a more attractive migration route for the lithium ions and the continuity of the skeleton promotes unimpeded migration of ions. Thus, it can be concluded that the implantation of p‐LGPS phase is the primary cause for the high$\;\sigma_{{\mathrm{Li}}^{+}}$ of the 3D composite electrolyte.

### 2.4 Compatibility with Lithium Metal

**Figure 5:**
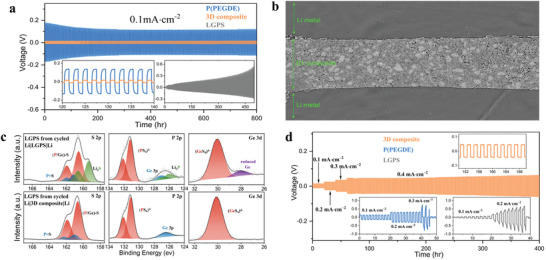
a) Lithium striping and plating curves of Li‐Li symmetric cells based on varied electrolyte of P(PEGDE), LGPS, and the 3D composite at room temperature. The area‐specific capacity is 0.1 mAh cm^−2^. b) Sliced images of Li|3D composite|Li symmetric battery after cycling. c) S 2p, P 2p and Ge 3d XPS of the cycled LGPS in the Li|LGPS|Li and Li|3D composite|Li symmetric batteries. d) Lithium striping and plating curves of Li—Li symmetric cells based on P(PEGDE), LGPS, and the 3D composite at varied current densities of 0.1, 0.2, 0.3, and 0.4 mAh cm^−2^.

Ensuring stable compatibility between electrolyte and lithium metal is a prerequisite for efficient operation of lithium metal batteries. To this end, we first performed electrochemical impedance tests and fitted impedance spectra for Li|Li symmetric cells based on different electrolytes (Figure S14, Supporting Information). Benefitting from the high $\sigma_{{\mathrm{Li}}^{+}}$ and the firm interfacial contact created by in situ polymerization, the 3D composite electrolyte exhibits an extraordinarily low ohmic impedance (375 Ω cm), as well as a satisfactory interfacial impedance (127 Ω cm^2^). Galvanostatic polarization experiments are then carried out at room temperature to evaluate the long‐term electrochemical compatibility of P(PEGDE), LGPS, and the 3D composite with Li by periodically charged and discharged for 1 h in Li|Li symmetric cells. For P(PEGDE), as shown in **Figure** **5**a, the Li|Li cell exhibits a large initial overpotential of 121 mV at a current density of 0.1 mA cm^−2^ due to the poor $\sigma_{{\mathrm{Li}}^{+}}$. By a huge contrast, this value is only 15 mV for Li|3D composite|Li cell, and flat voltage plateau remains stable for over 800 h, suggesting the better compatibility of 3D composite with lithium metal. The lithium‐ion transference number, in addition to the high $\sigma_{{\mathrm{Li}}^{+}}$, is a major contributor to this discrepancy. Based on the direct‐current polarization measurements (Figure S15, Supporting Information), the Bruce–Vincent–Evans equation calculates the lithium‐ion transference number of the 3D composite to be 0.5, which is substantially higher than that of the P(PEGDE) (0.28).^[^ ^40^ ^]^ To reveal the ability of the 3D composite to inhibit lithium dendrites, X‐ray computed tomography is conducted. Figure 4b displays the sliced images of Li|3D composite|Li symmetric battery after galvanostatic polarization. Obviously, the 3D composite maintains a clear boundary with Li metal after cycling. There is no indication of dendrites in the lithium metal phase, while no indication of lithium metal appears in the 3D composite phase. The surface morphology of Li metal before and after long‐term cycling are characterized by the SEM technique. As shown in Figure S16, Supporting Information, compared with the smooth lithium metal surface before cycling, the lithium metal surface after cycling demonstrates a distinct ion deposition pattern. It is clear that the deposition of lithium ions is flat and uniform, without undesirable roughness or any detectable dendrite structure. Therefore, the 3D composite can effectively inhibit the growth of dendrites and ensure the uniform deposition of lithium ions.

It should be noted though, that the initial polarization voltage of Li|LGPS|Li is only 7 mV; it increases continuously to 560 mV after 500 h. The EIS (Figure S17, Supporting Information) indicates the polarization process has been accompanied by an ongoing increment in impedance. Disassemble the battery, the original gray LGPS pellet is blackened absolutely after cycling for 200 h in Li|Li cell owing to side reactions (Figure S18, Supporting Information). Yet this similar phenomenon does not occur in the 3D composite electrolyte, which also contains LGPS components. It is hypothesized that P(PEGDE) serves a protective role in 3D composite, avoiding the reduction of LGPS by Li metal. In order to validate this speculation, X‐ray photoelectron spectroscopy (XPS) is conducted to investigate the surface compositions of LGPS and 3D composite after long‐term cycling.^[^ ^43^ ^]^ According to the comparison in Figure 5c, after cycling in Li|LGPS|Li, the Ge 3d spectrum exhibits a new peak of elemental germanium(≈28 eV), and other new peaks in S 2p and P 2p spectrum belong to Li_2_S and Li_3_P have also been detected, implying the reduction and decomposition of the LGPS.^[^ ^44^, ^45^ ^]^ Unsurprisingly the XPS spectra of the LGPS in the cycled Li|3D composite|Li do not appear the peaks regarding the reduction products and remain consistent with that of the original LGPS (Figure S19, Supporting Information). In conclusion, while porous skeleton for fast lithium‐ion transport contributes high $\sigma_{{\mathrm{Li}}^{+}}$ to 3D composite, the high‐voltage resistant P(PEGDE) matrix also provides protection to the p‐LGPS. It is this complementary relationship that allows the 3D composite to maintain comparative stability with lithium at a larger current density. Figure 5d demonstrates Li|3D composite|Li can cycle stably for 400 h even when the current density gradually rises to 0.4 mA cm^−2^. On the contrary, the P(PEGDE)‐based Li|Li symmetric cell remains stable only below 0.3 mA cm^−2^. The same situation occurs with the LGPS, where the increase in current density brings about a more severe decomposition, leading to a short circuit at 0.2 mA cm^−2^.

### 2.5 Electrochemical Performance of the 3D Composite Electrolyte Based ASLMB

**Figure 6:**
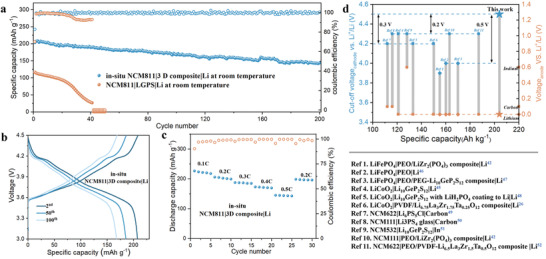
a) Cycling performance of in situ NCM811|3D composite|Li and NCM811|LGPS|Li ASLMBs working at a rate of 0.2 C. b) Galvanostatic charge‐discharge curves of in situ NCM811|3D composite|Li ASLB. c) The rate capability of NCM811|3D composite |Li ASLMB. d) The electrochemical performance of solid‐state batteries using different solid electrolytes and electrodes.^[^ ^24^, ^26^, ^42^, ^45^, ^46^, ^47^, ^48^, ^49^, ^50^, ^51^ ^]^

The electrochemical performance of NCM811|Li ASLMB is explored to verify the positive function of the synergistic strategy. As demonstrated in the previous section, the P(PEGDE)‐60 based NCM811|Li battery still performs charging and discharging behavior at a cut‐off voltage of 4.5 V. Nonetheless, due to the poor $\sigma_{{\mathrm{Li}}^{+}}$, the battery presents unsatisfactory discharge capacity and cycle performance. At a rate of 0.2 C, the initial discharge capacity of the P(PEGDE)‐60 based NCM811 battery is only 150.8 mAh g^−1^, and moreover, it has decayed by half even within 15 cycles (Figure S20, Supporting Information). For the in situ integrated NCM811|3D composite|Li ASLMB operated at 3.0–4.5 V versus Li^+^/Li, exceptional battery performance at room temperature is delivered with the boost of the high $\sigma_{{\mathrm{Li}}^{+}}$ p‐LGPS skeleton. As shown in **Figure** **6**a, an initial discharge specific capacity of 203.7 mAh g^−1^ is presented at the rate of 0.2 C, corresponding to an initial Coulombic efficiency of 83%. Even more remarkably, with an average Coulombic efficiency of over 99.6%, it still cycles stably for 200 cycles at 0.2 C and exhibited a discharge capacity of 144 mAh g^−1^ at the 200th cycle owing to the inevitable minor augment of polarization (Figure 6b). However, the LGPS with the high $\sigma_{{\mathrm{Li}}^{+}}$ cannot ensure qualified battery performance under the same circumstances. NCM811/LGPS/Li metal battery undergoes an abrupt capacity dwindle from 116 to nearly 28 mAh g^−1^ after 40 cycles due to the contact loss and significant polarization caused by the side reaction (Figure S21, Supporting Information).

The superior performance of the in situ integrated NCM811|3D composite|Li ASLMB is by no means due solely to the high $\sigma_{{\mathrm{Li}}^{+}}$ skeleton, and the interface compatibility that comes with in situ polymerization also plays an integral role. NCM811|3D composite|Li ASLMB is fabricated via ex situ processes for a fair comparison to confirm the importance of in situ integration. Without the optimization of the interface between electrolyte and electrode by in situ polymerization, the 3D composite‐based NCM811|Li ASLMB only exhibits a capacity of 172 mAh g^−1^. Worse still, after 20 cycles at a rate of 0.2 C, the battery capacity is only 75 mAh g^−1^, less than half of the initial capacity (Figure S22, Supporting Information). The poor cycling performance can be attributed to the inferior interfacial contact between cathode and electrolyte, which increases the interfacial impedance. This fact is further corroborated in the SEM imaging (Figure S23, Supporting Information). The ex situ polymerization fails to achieve complete contact between the 3D composite electrolyte and cathode, and a gap about 250 µm in length and 40 µm in width even appeases, which hinders ion transport. For in situ integrated NCM811|3D composite|Li, the liquid monomers crowd out the undesirable voids and the compact contact with cathode is preserved during solidification. The binder‐like effect integrates the cathode and electrolyte into a coherent entity, thus minimizing the interfacial impedance. With multiple advantages, the in situ NCM811|3D composite|Li shows splendid rate performance with a discharge capacity of 219 mAh g^−1^ at 0.1 C, 179 mAh g^−1^ at 0.3 C, 163 mAh g^−1^ at 0.4 C, and 136 mAh g^−1^ at 0.5 C (Figure 6c), respectively. Compared to the previously reported solid‐state batteries based on various electrolytes and electrodes (Figure 6d), the in situ NMC811|3D composite|Li ASLMB has significant advantages regarding both working voltage and discharge capacity.^[^ ^24^, ^26^, ^42^, ^45^, ^46^, ^47^, ^48^, ^49^, ^50^, ^51^ ^]^ It is widely acknowledged that efforts to improve working cut‐off voltage and specific capacity aim at acquiring high energy density. The electrochemical performance suggests in situ polymerization of salt‐concentrated PEGDE implanted with p‐LGPS skeleton is a feasible and promising strategy for the application to high energy density ASLMBs.

## 3 Conclusion

In summary, we propose a synergistic strategy and prepare a 3D composite electrolyte through in situ polymerizing salt‐concentrated PEGDE monomers implanted with porous skeleton (p‐LGPS). The high concentration of P(PEGDE) significantly enhances its antioxidant properties through the coordination of EO segments with more Li^+^, while the 3D backbone structure of p‐LGPS provides a rapid and coherent transport path for Li^+^. After organic‐inorganic compounding, the 3D composite exhibits an $\sigma_{{\mathrm{Li}}^{+}}$ of 7.7 × 10^−4^ S cm^−1^ and a wide electrochemical window of up to 5 V. In addition, together with the integration effect of in situ polymerization on the electrolyte/electrode interface, ASLMBs of NCM|Li demonstrates high discharge capacity of 204 mAh g^−1^ and cycle performance of up to 200 cycles, which perfectly meets the needs of high‐energy‐density batteries. This paper proposes a reliable method to compensate for the shortcomings of solid electrolytes in terms of voltage, conductivity, etc., and provides a valuable reference for the practical application of safe lithium batteries.

## 4 Experimental Section

To prepare p‐LGPS, LGPS powders were firstly synthesized via solid reaction processes. Specifically, stoichiometric Li_2_S (Alfa Aesar, 99.9%), P_2_S_5_ (MACKLIN, ≥99%), and GeS_2_ (Aladdin, 99.999%) were added into a tightly sealed zirconia pot filled with zirconia balls of 10 mm in diameter and ball‐milled at a speed of 400 rpm for 40 h. Then, the mixture was pressed into tablets and transferred into a quartz tube, which was then sealed and sintered at 600 °C for 5 h. After slowly cooling to room temperature, the tablets were taken out of the tube and manually ground to acquire LGPS powers. SeS_2_ (Aladdin, 97%) and the as‐prepared LGPS powders were mixed according to the weight ratio of 1:1. Then 25 mg of the mixture was pressed at 300 MPa in a *φ*10 mm mold to obtain green pellets with a thickness of 150 um, which were then transferred to a quartz tube and sintered at 500 °C for 5 h, during which the SeS_2_ was volatilized to leave the pellets porous, obtaining p‐LGPS. Due to the vulnerability of sulfides to moisture, the entire preparation process was carried out under the protection of argon.

The 3D composite cells were assembled by in situ and ex situ methods, respectively, to demonstrate the excellent interfacial compatibility performance of the in situ integration strategy by comparing the electrochemical performance. In order to prepare the in situ ASLMB, the 40 µL PEGDE monomer precursors were instilled into the porous tablet placed on the lithium anode. Next, the coin cell was assembled after cathode@Ti foil was tightly attached to the electrolyte, and put it in an 80 °C oven for 24 h to accomplish in situ polymerization. Correspondingly, the composite tablet filled with 40 µL monomer precursors was continuously heated at 80 °C for 24 h, after polymerization, clamped with lithium anode and cathode@Ti foil to complete the ex situ battery assembly.

Other experiments can be found in the Supporting Information.

## Conflict of Interest

The authors declare no conflict of interest.

## Supporting information

Supporting Information

## Data Availability

The data that support the findings of this study are available from the corresponding author upon reasonable request.
