# Supplementary material for: Percolated Sulfide in Salt‐Concentrated Polymer Matrices Extricating High‐Voltage All‐Solid‐State Lithium‐metal Batteries
Source: Adv Sci (Weinh). 2022 Jun 24;9(25):2202474. doi: 10.1002/advs.202202474 (PMC9443466; doi:10.1002/advs.202202474)
Supplement: Supplementary file 1 — Supporting Information [file ADVS-9-2202474-s001.pdf]

ADVANCED SCIENCE
Open Access

Supporting Information

for *Adv. Sci.*, DOI 10.1002/advs.202202474

Percolated Sulfide in Salt-Concentrated Polymer Matrices Extricating High-Voltage All-Solid-State Lithium-metal Batteries

Feng Jiang, Yantao Wang, Jiangwei Ju, Qian Zhou, Longfei Cui, Jinzhi Wang, Guoxi Zhu, Huancheng Miao, Xinhong Zhou* and Guanglei Cui**

Supporting Information

Percolated sulfide in Salt-concentrated polymer matrices extricating high-voltage all-solid-state lithium-metal batteries

Feng Jiang, Yantao Wang, Jiangwei Ju, Qian Zhou, Longfei Cui, Jinzhi Wang, Guoxi Zhu, Huancheng Miao, Xinhong Zhou* and Guanglei Cui**

Experimental

Preparation of cathode

The $LiNi_{0.8}Mn_{0.1}Co_{0.1}O_2$ (NCM811) cathode composites are prepared as follows: the cathode slurry is firstly prepared by grinding 80 wt % NCM811 powders, 10 wt % polyvinylidene fluoride (PVDF) binder and 10 wt % Super P in N-methyl-2-pyrrolidone (NMP) agent. The slurry is then casted on a clean Titanium foil and dried in vacuum oven at 60 °C. After dried for 24 hrs, the NCM811 cathode@Ti foil is taken out and punched into disks in diameters of 10 mm. The loading of the active material is 2.4 ± 0.1 mg cm^{-2}.

Battery assembly

Steel|steel symmetric batteries are fabricated for ionic conductivity test. Symmetric steel| $Li_{10}GeP_2S_{12}$ (LGPS)|steel battery is fabricated by pressing 50 mg LGPS powders in one 10 mm diameter mold. To fabricate the steel|p-LGPS|steel symmetric battery, carbon paste is evenly brushed onto each side of p-LGPS and dried naturally. Then the p-LGPS with carbon paste is sandwiched between two steel discs to complete the assembly of coin cell.

To fabricate steel|3D composite|steel and steel|P(PEGDE)|steel symmetric batteries, PEGDE monomer precursors are firstly prepared by dissolving 0.6 g LiTFSI into 1 g PEGDE to acquire Li^+

concentration of 3 M. Before used, 0.015 g LiDFOB is added into the solution as polymerization initiator. p-LGPS or cellulose separator are placed on one steel disc, then 40 μL of the PEGDE solution is injected into p-LGPS or cellulose separator followed by the placement of another steel disc on them to complete the assembly of coin cells.

Li|Li symmetric batteries are used for galvanostatic polarization test. The fabrication processes of symmetric Li|3D composite|Li and Li|P(PEGDE)|Li batteries are the same as the method used in steel|steel symmetric batteries, except that the steel discs are replaced by lithium foil. To fabricate Li|LGPS|Li symmetric batteries, 50 mg of the LGPS powder is pressed at 300 MPa in a ϕi10 mm mold to obtain green pellets with a thickness of 300 um, which are then sandwiched between two lithium foil to complete the assembly of coin cell.

Steel|Li asymmetric batteries are used for linear scan voltammetry (LSV) and cyclic voltammetry (CV) test. The fabrication processes of asymmetric steel|3D composite|Li and steel|P(PEGDE)|Li batteries are the same as the method used in steel|steel symmetric batteries, except that one steel disc is replaced by lithium foil.

The 3D composite cells are assembled by in situ and ex situ methods, respectively, to demonstrate the excellent interfacial compatibility performance of the in situ integration strategy by comparing the electrochemical performance. In order to prepare the in situ ASLMB, the 40 μl PEGDE monomer solution is instilled into the porous tablet placed on the lithium anode. Next, the coin cell is assembled after cathode@Ti foil is tightly attached to the electrolyte, and put it in an 80 °C oven for 24 hrs to accomplish in situ polymerization. Correspondingly, the composite tablet filled with 40 ul monomer solution is continuously heated at 80 °C for 24 hrs, after polymerization, clamped with lithium anode and cathode@Ti foil to complete the ex situ battery assembly.

NCM811|LPSCl|Li ASLB: The NCM811 and LGPS powders are fully mixed and ground according to the weight ratio of 7:3 to fabricate the NCM811 cathode composite. 80 mg LGPS powder is pressed in a poly(ether-ether-ketone) (PEEK) mold with an inner diameter of 10 mm at a pressure of

200 MPa, and then 10 mg NCM811 cathode composite is uniformly spread on one side of the LGPS electrolyte layer and compressed at 350 MPa. Finally, a piece of lithium foil is attached to the other side of the LGPS layer with a pressure of 30 MPa. Leave the battery for 6 hrs before testing.

All the above assembly processes are performed in an argon filled glove box (O_2 < 0.01 ppm; H_2O < 0.01 ppm).

Materials Characterizations

XRD patterns are collected using an X-ray diffractometer (Rigaku SmartLab) equipped with a Cu Kα radiation source. SEM images are characterized using Hitachi S-4800 equipped with EDS. FTIR spectra measurements are conducted on a Fourier transform infrared spectrometer (Bruker VERTEX 70). Nuclear magnetic resonance (NMR, Bruker AVANCE III 600 MHz) is used to analyze the molecular structures of polymers. Raman spectra are acquired using a micro-Raman spectrometer (Renishaw, inVia). XPS measurements are conducted by an ESCALab 250Xi (Thermo Scientific) spectrometer equipped with an Al Kα achromatic X-ray source. ^{6}Li magic angle spinning (MAS) solid-state NMR is performed on a 20.0 T (850 MHz) Varian VNMRS spectrometers with a ^{6}Li Larmor frequency of 125 MHz. P(PEGDE), LGPS, and the 3D composite samples are packed in 4.0 mm rotors and spun at a speed of 8 kHz. For ^{6}Li NMR, the 90° pulse length was 5 µs. 8 scans are acquired for LGPS with a recycle delay of 20 s, and 64 scans are acquired for the 3D composite and P(PEGDE) with a recycle delay of 600 s. Chemical shifts are referenced to solid LiCl at 0 ppm.

MD Simulation

In this study, all molecular dynamics (MD) simulations were determined using the Forcite module in the Materials Studio package. Three MD simulation models containing molecules P-PEGDE, LiTFSI with a different mole ratio

The initial mole ratio of each component in the four simulation systems is in strict agreement with our experimental data. After energy minimization to remove potential overlaps among all

molecules, all simulations were equilibrant at constant pressure (1 atm) and temperature (298 K) dynamics for about 10 ns. Atomic coordinates were saved for every 1000 steps (Time step = 1.0 fs). The analysis was performed by the final 5 ns of each trajectory.

During the simulation, periodic boundary conditions were applied in all three dimensions.

The MD simulations of the three systems were performed after potentials assigned to each atom. The long-range electrostatic interactions have been accounted for using the Ewald method. The total energy is written as a combination of valence terms including diagonal and off-diagonal cross-coupling terms and nonbond interaction terms, which include the Coulombic and Lennard-Jones functions for electrostatic and van der Waals interactions,

$$E = E_{\mathrm{bonds}} + E_{\mathrm{angles}} + E_{\mathrm{dihedrals}} + E_{\mathrm{cross}} + E_{\mathrm{VDW}} + E_{\mathrm{elec}} \qquad (1),$$

where E_{VDW} and E_{elec} are given by the eq 2:

$$E_{\text{non-bond}} = E_{\mathrm{VDW}} + E_{\mathrm{elec}} = \sum \varepsilon_{ij} \left[2\left(\frac{\sigma_{ij}}{r_{ij}}\right)^9 - 3\left(\frac{\sigma_{ij}}{r_{ij}}\right)^6 \right] + \sum \frac{q_i q_j}{r_{ij}} \qquad (2),$$

The parameters for each like-site interaction are given by the COMPASSII force field.[1] The energies of the initial configurations were minimized with the Smart Minimizer method. After that, the typical structures of each solution were selected from the simulation trajectory. All structures are optimized by and the orbital energy gap was calculated Geometry optimization and Orbital energy gap calculations were carried out with the xtb software[2] in GFN2-xTB method.[3]

Electrochemical Measurements

The ionic conductivity is calculated according to EIS tests in the frequency range from 7 MHz to 1 Hz with an applied amplitude of 10 mV. The transference number is tested by applying a constant voltage to a symmetrical battery until the current reaches a stable value and determined based on Equation 3:

$$t_{Li^+} = \frac{I^{ss} R_{ohm}^{ss} (\Delta V - I^o R_P^o)}{I^o R_{ohm}^o (\Delta V - I^{ss} R_P^{ss})} \quad (3),$$

where ΔV is the applied amplitude voltage, 10 mV, and the superscripts of *o* and *ss* represent the initial and steady state, respectively. *I* is the current. R_{ohm} and R_p are the ohmic and interfacial resistance of the symmetrical batteries, respectively. The DC polarization tests of Li|Li symmetrical batteries are conducted at a current density of 0.1 mA cm^{-2} for 800 hrs. The CV tests are conducted between −1.0 and 5.0 V at a scanning rate of 0.1 mV s^{-1} at room temperature. Galvanostatic cycling tests are conducted using a Land battery test system (Land CT2001A, Wuhan Land Electronic Co., Ltd., China) under various current densities at room temperature.

Table S1. Specific information on PEGDE-20, PEGDE-40, and PEGDE-60

	LiTFSI content (wt %)	Concentration in monomer solution (mol/L)	EO/Li rate
P(PEGDE)-20	20	1	27:1
P(PEGDE)-40	40	2	27:2
P(PEGDE)-60	60	3	9:1

Table S2. EIS fitting results for the Li|3D composite|Li, Li|P(PEGDE)|Li and Li|LGPS|Li symmetric cells.

	R_{ohm} (Ω cm)	R_{int} (Ω cm^2)	C_p (F)
Li|3 D composite|Li	375	127	8.5×10^{-6}
Li|PEGDE|Li	229545	769	8.4×10^{-6}
Li|LGPS|Li	95	45	3.9×10^{-8}

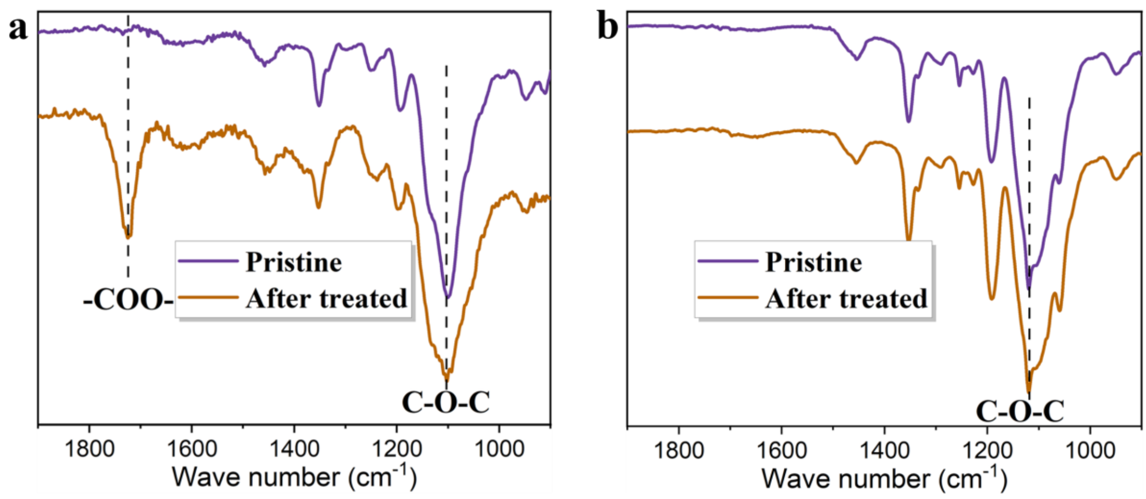

Figure S1. FTIR spectra of (a) P(PEGDE)-20 and (b) P(PEGDE)-60 on the cathode side before and after the EFA test.

As shown in **Figure S1**, the FTIR spectrum of P(PEGDE)-20 after EFA testing shows a new characteristic peak at 1725 cm^{-1}, which is attributed to the formation of esters species formed by the oxidation and decomposition of EO segments at high voltage. For P(PEGDE)-60, the spectra before and after the EFA test are highly consistent, suggesting that P(PEGDE)-60 can remain stable at 4.7 V. It is worth noting that compared to the pristine P(PEGDE)-20, the pristine P(PEGDE)-60 appears stronger characteristic peaks at 1058 cm^{-1}, 1293 cm^{-1}, 1353 cm^{-1}, etc., which are caused by different LiTFSI contents.

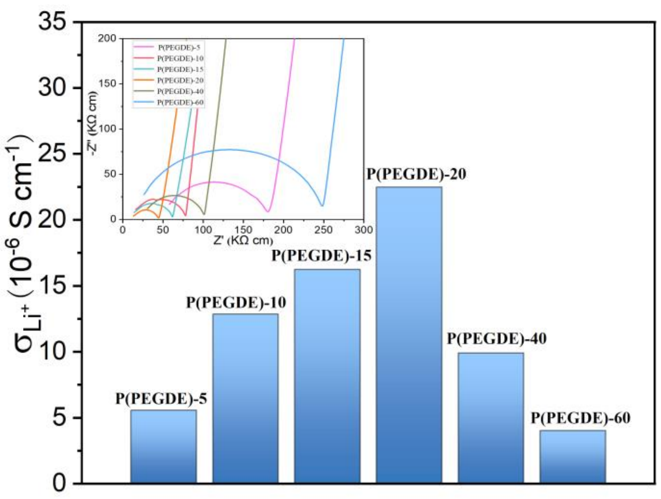

Figure S2. Ionic conductivity of P(PEGDE) with different LiTFSI concentrations at room temperature.

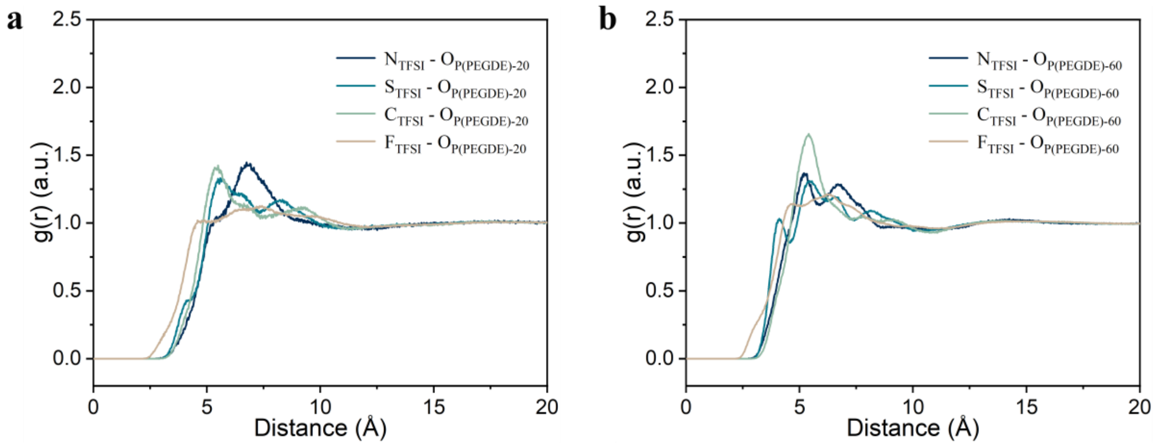

Figure S3. RDF between atoms in $TFSI^-$ and O atoms in (a) P(PEGDE)-20 and (b) (PEGDE)-60.

Accoring to MD simulation trajectory, the same RDF calculation was performed for the O atom in P(PEGDE) and the other atoms in LiTFSI. The RDF results show that distance between $TFSI^-$ and the O atom is generally beyond 3 Å, while no obvious characteristic peaks appear. It can be concluded that $TFSI^-$ and P(PEGDE) are distributed in a chaotic state and do not interact with each other.

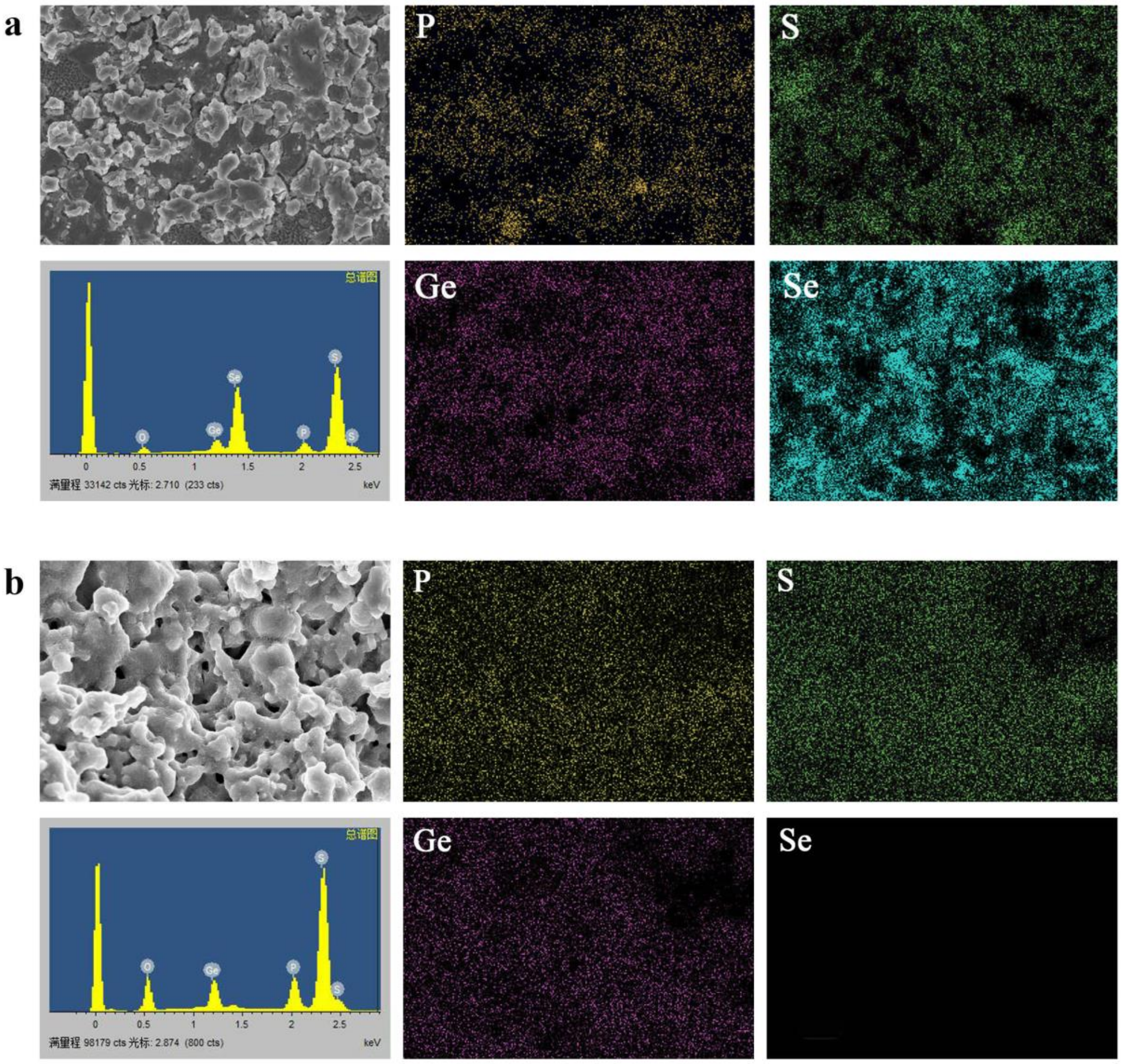

Figure S4. EDS mapping of (a) the mixture of LGPS and SeS_2 and (b) p-LGPS.

According to the EDS results, it can be observed that while the coherent pore structure is successfully prepared, the high temperature sintering also results in the complete volatilization of SeS_2. The oxygen is due to air contamination during sample transfer.

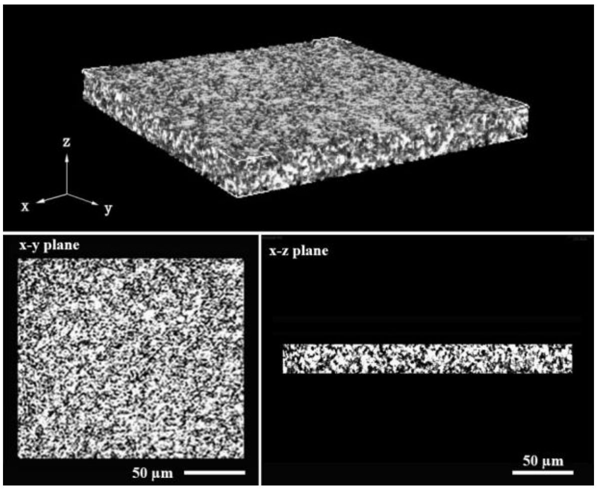

Figure S5. The 3D reconstruction image of p-LGPS and corresponding 2D sliced images from the x-y, x-z plane.

To further reveal the porous microstructure of p-LGPS, the X-ray computed tomography is conducted. The gray and black represent LGPS phase and pores, respectively. The uniform pore distribution observed in both the 3D reconstruction image of p-LGPS and corresponding 2D sliced images from x-y, x-z plane proves p-LGPS with percolated porous structure has been fabricated successfully.

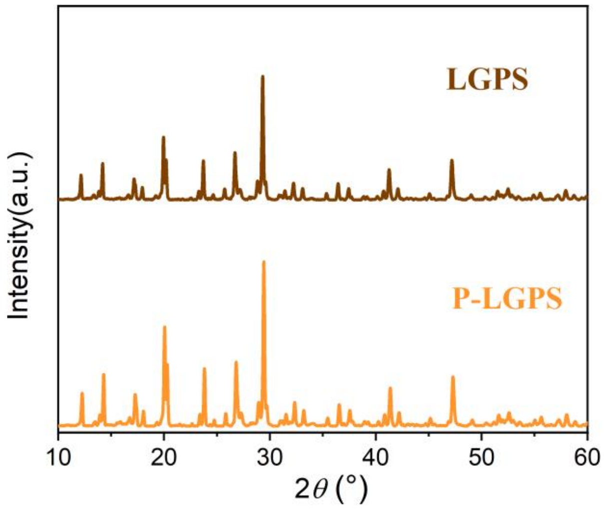

Figure S6. XRD patterns of LGPS and the p-LGPS.

As shown in **Figure S6**, the as-synthesized LGPS powders are well crystallized verified by XRD. The XRD pattern of P-LGPS is well consistent with that of the as prepared powders, implying that the crystal structure of LGPS does not change and all the SeS_2 has been removed during the pore-forming process.

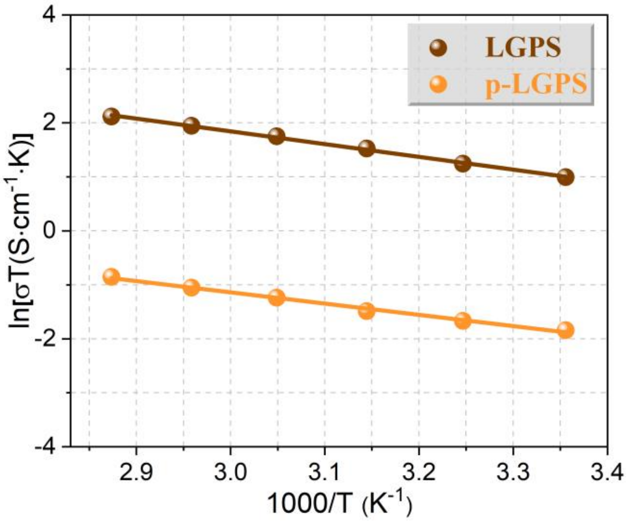

Figure S7. Conductivity comparison of the as-prepared LGPS and p-LGPS under varied temperatures.

Figure S7 displays the comparison of the ionic conductivities between the as-prepared LGPS and p-LGPS under different temperatures. The ionic conductivity of p-LGPS can reach as high as about 4.8 $\times$ 10^{-4} S cm^{-1} at room temperature, which is 19 times smaller than that of the dense LGPS due to the large porosity of p-LGPS.

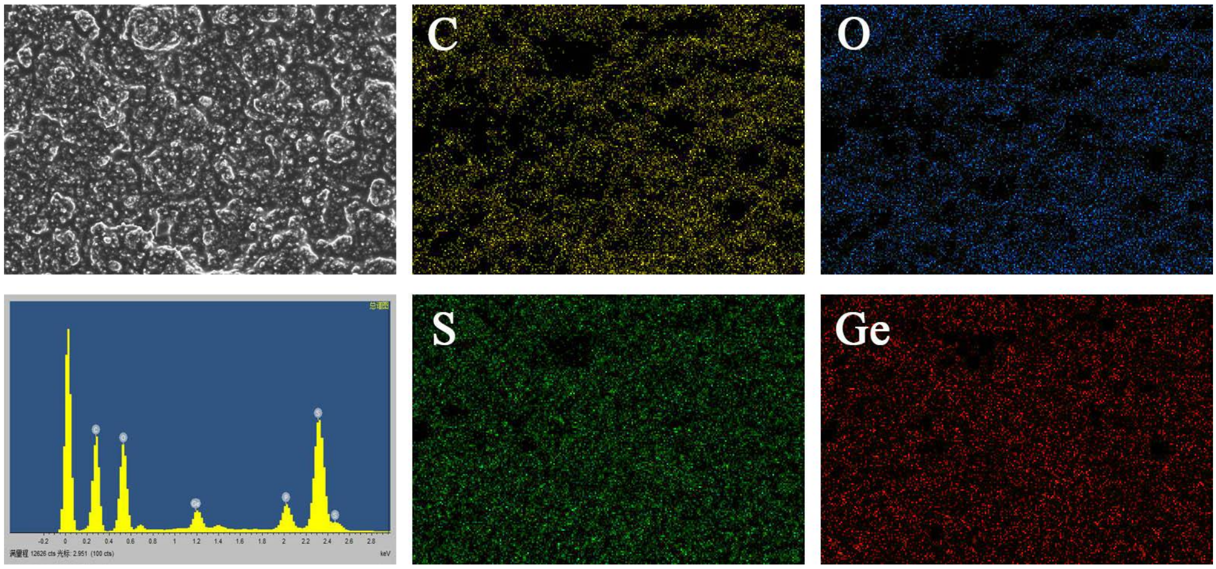

Figure S8. EDS mapping of the 3D composite.

As shown in **Figure S8**, the EDS mapping of the 3D composite demonstrates the homogeneous distribution of both organic and inorganic components.

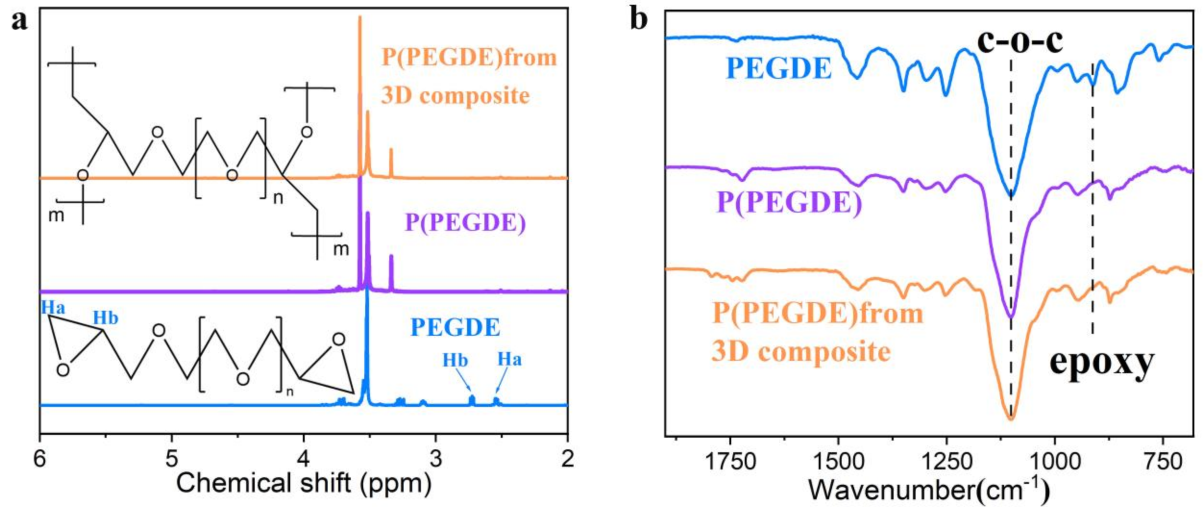

Figure S9. (a) ^{1}H NMR spectra of PEGDE, pristine P(PEGDE) and P(PEGDE) from the 3D composite using deuterated dimethyl sulfoxide as solvent. (b) FTIR spectra of PEGDE, pristine P(PEGDE), and P(PEGDE) from the 3D composite.

As shown in **Figure S9**, the disappearance of the peaks at 2.72 ppm and 2.52 ppm attributed to hydrogen atoms in the epoxy ethyl group marked by Ha and Hb in the ^{1}H NMR spectra, as well as the disappearance of the peak at 917 cm^{-1} attributed to epoxy in the FTIR spectra, validate that PEGDE is successfully polymerized regardless of the presence of p-LGPS.

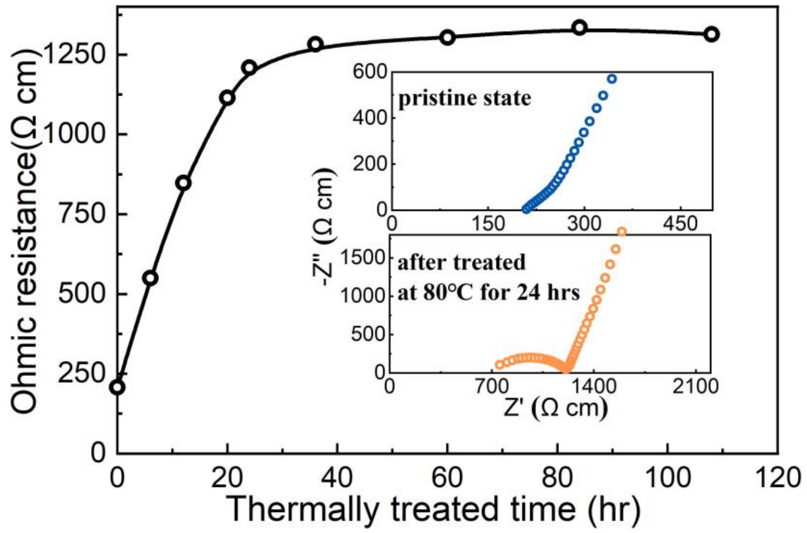

Figure S10. Thermal evolution of ohmic resistance at 80 °C for steel|3D composite|steel symmetrical cells. Inset shows the room-temperature EIS plots of the steel|3D composite|steel symmetrical cells before and after thermal treatment at 80 °C for 24 hrs.

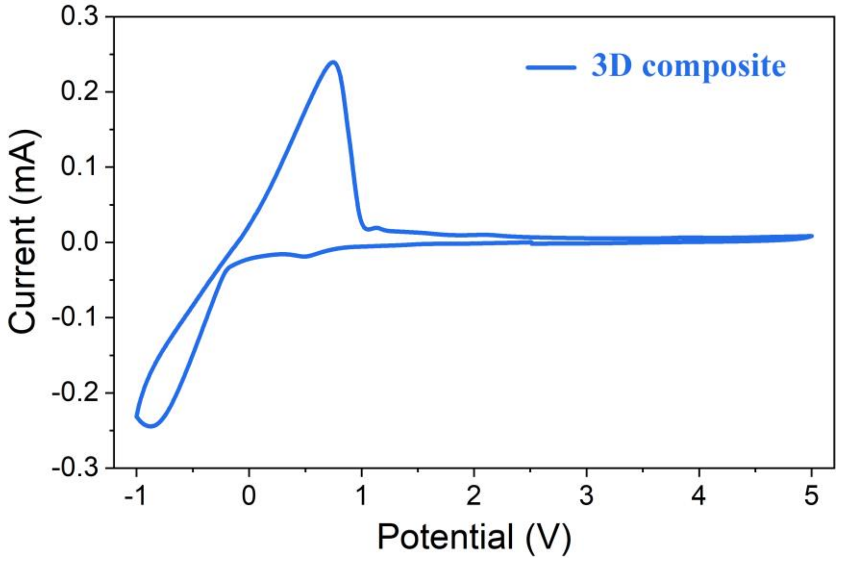

Figure S11. CV profiles for 3D composite at 0.1 mV s^{-1} between −1.0−5.0 V at room temperature.

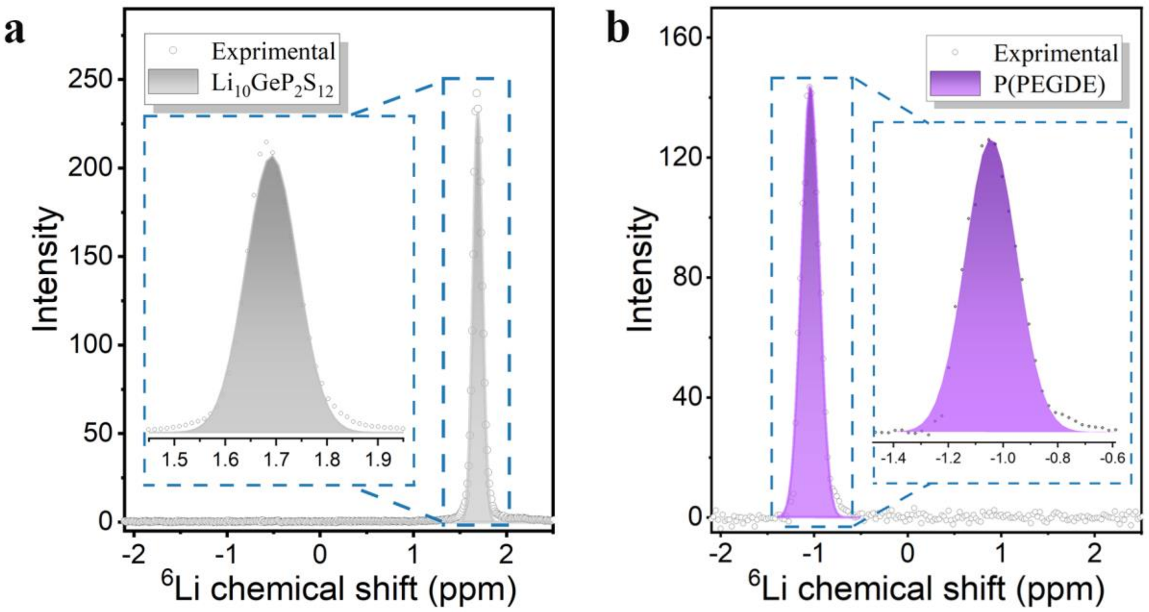

Figure S12. ^{6}Li SSNMR spectra of the pristine (a) LGPS and (b) P(PEGDE).

SSNMR is firstly performed on pristine P(PEGDE)-60 and LGPS to provide a reference for the characteristic peaks of the ^{6}Li signal in different local environments. ^{7}Li and ^{6}Li are both NMR-active isotopes, and using an isotope-replacement method, the source of ionic conductivity and Li ion migration behavior in 3D composites can be elucidated by solid-state NMR (SSNMR). More importantly, SSNMR is sensitive to the local structural environment and thus can be used to distinguish lithium ions in the polymer phase, sulfide phase, and polymer-sulfide interface in

composite electrolytes, making SSNMR an effective vehicle for researching the local structural environment and dynamics of lithium ions. To this end, a Li-Li symmetric cell is prepared by using a composite electrolyte and a ^{6}Li metal electrode. Unidirectional constant-current polarization of the cell at a current density of 60 μA cm^{-2} allows $^{6}Li^{+}$ ions to move through the composite electrolyte from one electrode to the other electrode, driven by the potential. During the polarization process, $^{6}Li^{+}$ replaces the $^{7}Li^{+}$ in the composite electrolyte, leaving diffusion paths. By comparing the ^{6}Li spectra of the composite electrolyte before and after polarization, an increase in the intensity of the ^{6}Li peak can be observed in the local environment where the ^{6}Li ions pass, while the evolution of the ^{6}Li content is more significant for the paths where the migration of Li^{+} is relatively favorable, thus revealing the complex Li ion migration in the composite electrolyte.、

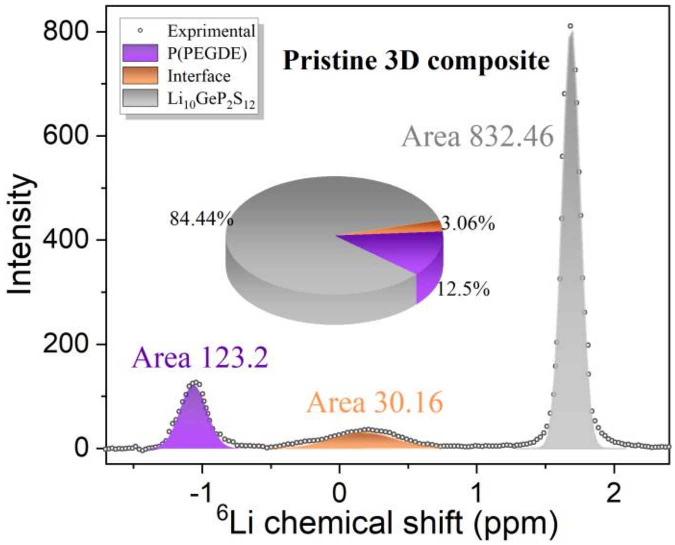

Figure S13. Quantitative fitting of the 3D composite ^{6}Li SSNMR spectra before polarization.

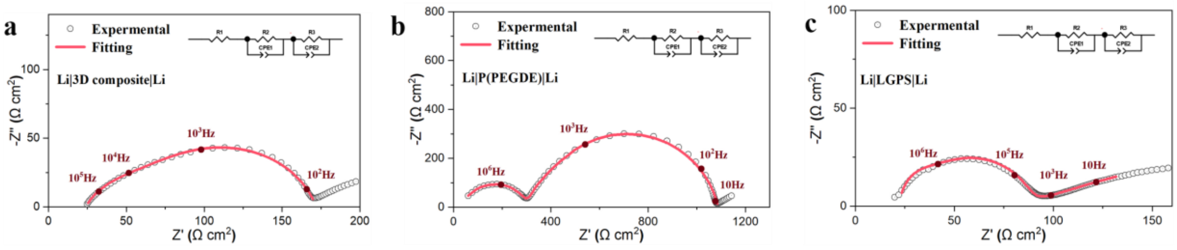

Figure S14. EIS plots and equivalent circuit models of Li-Li symmetrical cells based on (a) 3D composite, (b) PEGDE and (c) LGPS. The characteristic frequencies are marked.

Li-Li symmetrical cells based on 3D composite and PEGDE are both in situ polymerized by heating at 80 °C for 24 hrs. The EIS plots consist of a semicircle and a small tail, where the intercept of the semicircle with the real axis represents the ohmic impedance and the span of the semicircle represents the interfacial impedance. The exception is the EIS plot of Li|P(PEGDE)|Li cell consisting of two semicircles, in which case the ohmic impedance is determined by the span of the high frequency semicircle, while the interface is determined by the span of the intermediate frequency semicircle. The fitting results are summarized in **Table S2**.

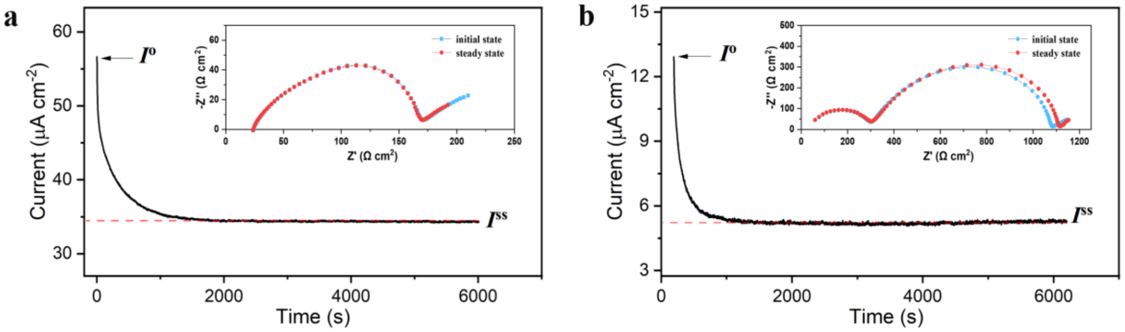

Figure S15. Current variation with time decay during polarization of (a) Li|3D composite|Li and (b) Li|P(PEGDE)|Li configuration at an applied voltage of 10 mV; the inset shows the impedance comparison before and after polarization.

To reduce the polarization and suppress the formation of lithium dendrites, a high Li^+ transference number is critical. Via a galvanostatic polarization method, Li^+ transference number of the 3D composite is estimated to be 0.5, which is much higher than that of the P(PEGDE), 0.28. The high Li^+ transference number may be attributed to the large fraction of LGPS.

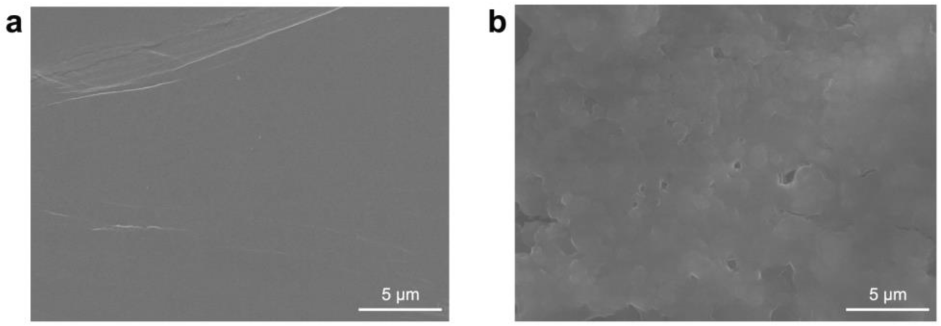

Figure S16. Typical top-view SEM image of Li metal a) before and b) after Li plating/stripping 800 h in Li|3D composite|Li symmetric battery.

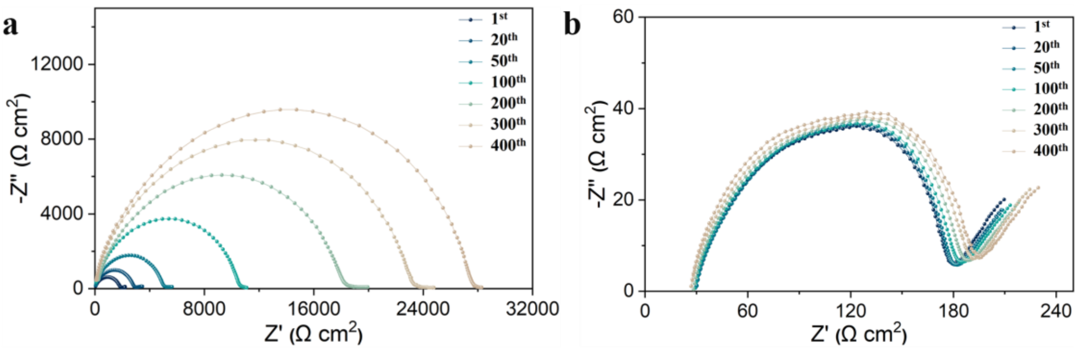

Figure S17. Impedance evolution of (a) Li|LGPS|Li and (b) Li|3D composite|Li symmetric batteries with varied cycle at 0.1 mA^{-2}.

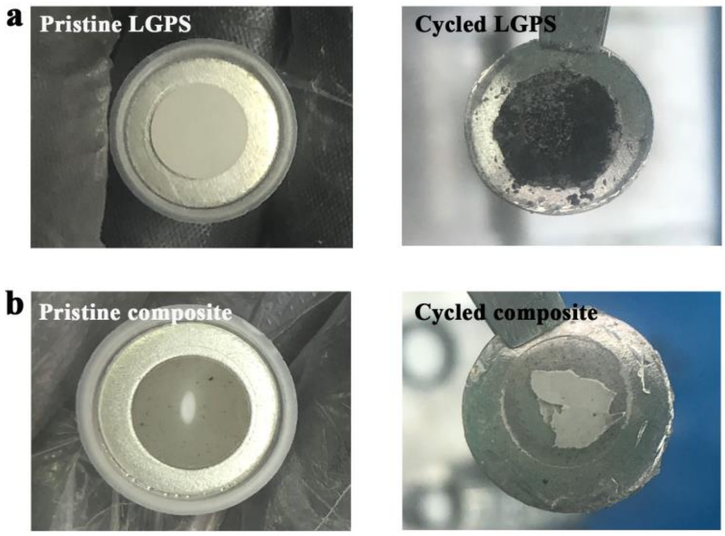

Figure S18. Digital images of the pristine and cycled LGPS in the (a) Li|LGPS|Li and (b) Li|3D composite|Li symmetric batteries.

It should be noted here the strong adhesion between electrolyte and electrode caused by in situ polymerization lead to unavoidable damage to the 3D composite when disassembling the cell.

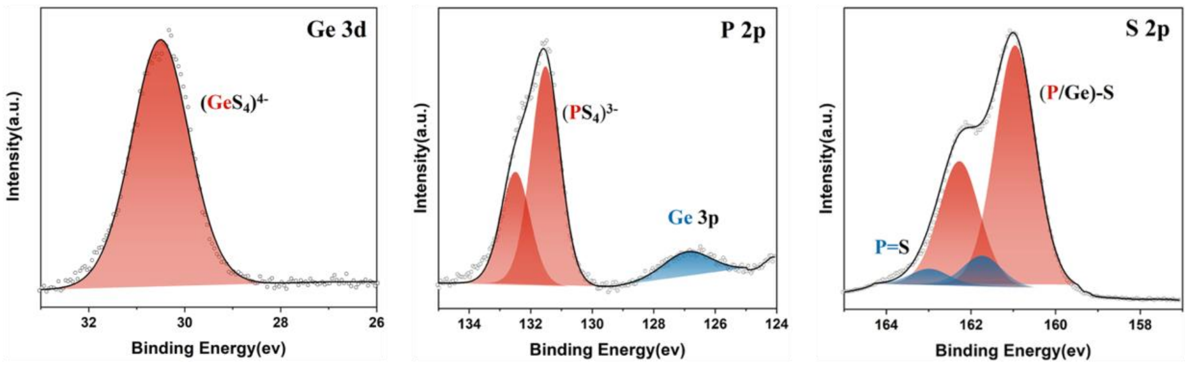

Figure S19. Ge 3d, P 2p and S 2p XPS spectra of the pristine LGPS

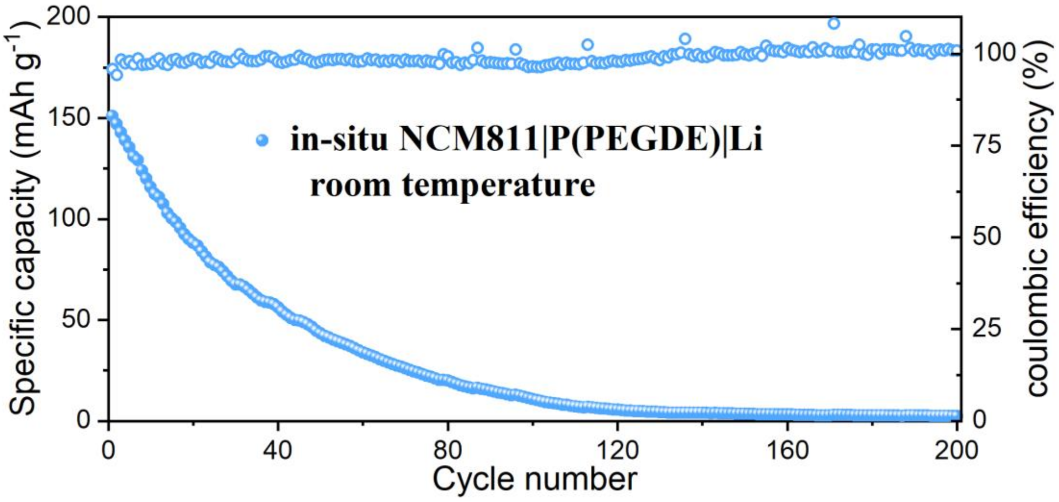

Figure S20. Cycling performance of in situ NCM|P(PEGDE)|Li battery at a rate of 0.2 C.

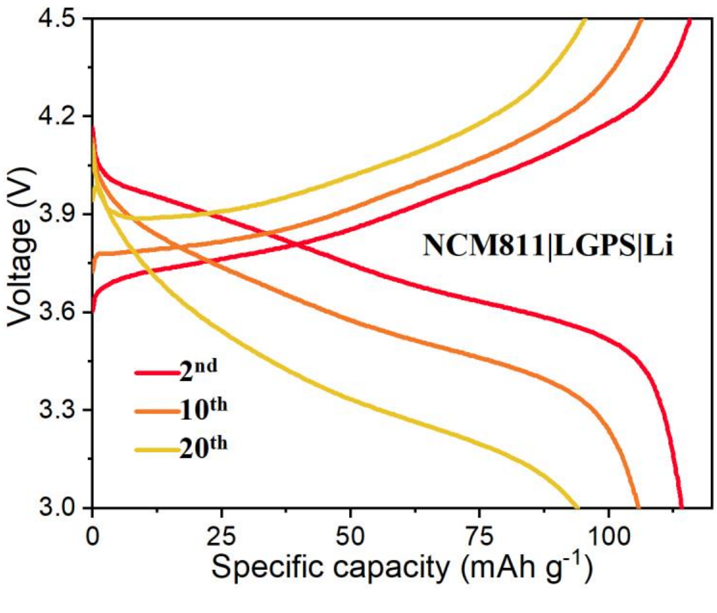

Figure S21. Galvanostatic charge-discharge curves of NCM811|LGPS |Li ASLMB.

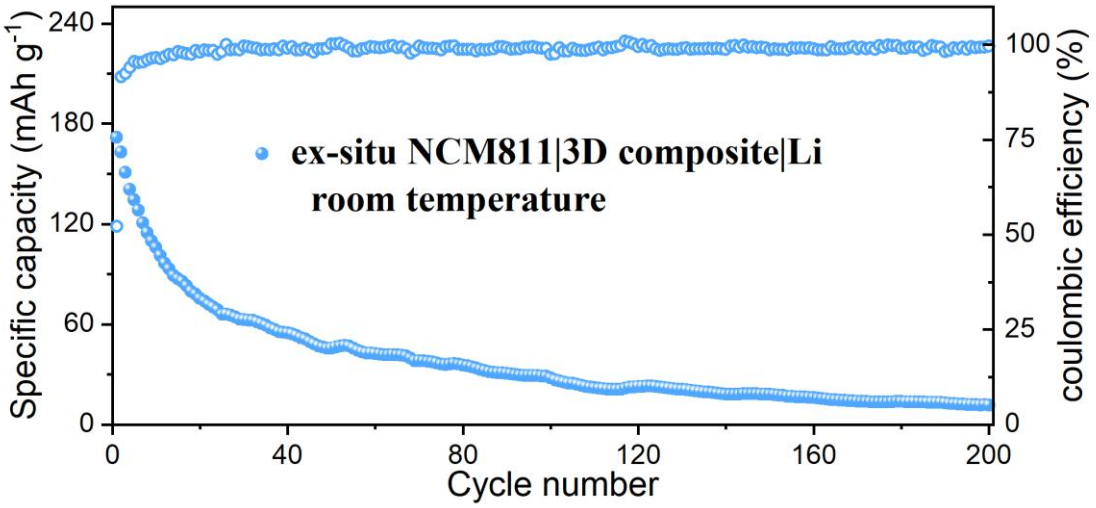

Figure S22. Cycling performance of ex situ NCM|3D composite|Li battery at a rate of 0.2 C.

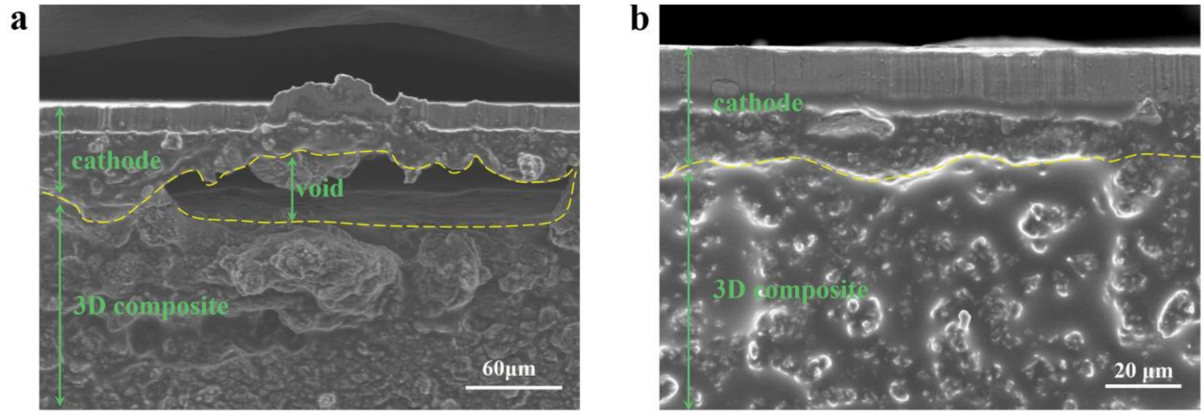

Figure S23 Typical SEM cross-sectional view of the cathodic interface from the disassembled (a) ex situ and (b) in situ NCM811|3D composite|Li ASLBs.

References

[1] H. Sun, Z. Jin, C. Yang, R. Akkermans, S. Robertson, N. Spenley, S. Miller, S. Todd, *Journal of Molecular Modeling* **2016**, 22, 47.

[2] C. Bannwarth, E. Caldeweyher, S. Ehlert, A. Hansen, P. Pracht, J. Seibert,S. Spicher, S. Grimme, *WIREs Computational Molecular Sci*ence **2021**, 11, e1493.

[3] C. Bannwarth, S. Ehlert, S. Grimme, *Journal of Chemical Theory and Computation*. **2019**, *5*, 1652.
